# Case report: Improvement in refractory functional seizures, depression, and quality of life with ketamine-assisted therapy

**DOI:** 10.3389/fnins.2023.1197409

**Published:** 2023-06-12

**Authors:** Elena Argento, Egiroh Omene, Alexandria H. Jaeger, Angela Kertes, Kaitlyn A. Mitchell, Candace Necyk, Paul Thielking, Evan Cole Lewis

**Affiliations:** ^1^Numinus Wellness Inc., Vancouver, BC, Canada; ^2^Faculty of Medicine, British Columbia Centre on Substance Use, University of British Columbia, Vancouver, BC, Canada; ^3^Neurology Centre of Toronto by Numinus, Toronto, ON, Canada; ^4^North Toronto Psychology, Toronto, ON, Canada; ^5^Numinus Wellness, Draper, UT, United States

**Keywords:** functional seizures, functional neurological disorder, ketamine-assisted therapy, mental health, quality of life

## Abstract

Functional seizures, a primary subtype of functional neurological disorder (FND), are a known cause of serious neurological disability with an increasing awareness of their impact amongst the neuroscience community. Situated at the intersection of neurology and psychiatry, FND is characterized by a range of alterations in motor, sensory or cognitive performance, such as abnormal movements, limb weakness, and dissociative, seizure-like episodes. Functional seizures are known, in part, to have psychological underpinnings; however, the lack of effective and consistent treatment options requires research and novel approaches to better understand the etiology, diagnosis and what constitutes a successful intervention. Ketamine, a selective blocker of the N-methyl-D-aspartate receptor, has a well-established safety and efficacy profile. In recent years, ketamine-assisted therapy has shown increasing potential for treating a broad range of psychiatric conditions, building on its demonstrated rapid-acting antidepressant effects. Here we present a 51-year-old female with refractory daily functional seizures leading to significant disability and a medical history significant for major depressive disorder (MDD) and posttraumatic stress disorder (PTSD). After unsuccessful treatment attempts, the patient underwent a novel protocol with ketamine-assisted therapy. After 3 weeks of ketamine-assisted therapy followed by 20 weeks of intermittent ketamine treatment and ongoing integrative psychotherapy, the patient’s seizures were significantly reduced in frequency and severity. She experienced significant improvements in depressive symptoms and functional ability scores. To our knowledge, this is the first reported case describing improvement in functional seizures following ketamine-assisted therapy. While rigorous studies are needed, this case report encourages further investigation of ketamine-assisted therapy for functional seizures and other functional neurological symptoms.

## Introduction

Functional neurological disorder (FND) is a known cause of serious neurological and physical disability, resulting in increased interest amongst the neuroscience community. FND is characterized by a range of neurological symptoms and alterations in motor, sensory or cognitive performance, such as abnormal movements, limb weakness, and dissociative, seizure-like episodes known as functional seizures (FS) or psychogenic nonepileptic seizures (PNES; Espay et al., 2018; Drane et al., 2020). These experiences are distressing and have a significant impact on quality of life. Outcomes are generally poor and include a mortality rate 2.5 times higher than the general population (Goldstein et al., 2020; Nightscales et al., 2020). Healthcare costs associated with FND are substantial: United States Emergency Department and inpatient costs are estimated at $1.2 billion dollars a year (Stephen et al., 2021).

Functional seizures, a primary subtype of FND, are paroxysmal events that resemble epileptic seizures but are not associated with electroencephalographic (EEG) epileptiform correlates. Current models propose that FS are a disorder of brain networks, with psychological, biological and social predisposing, precipitating, and perpetuating factors (LaFrance et al., 2014; Brown and Reuber, 2016; Goldstein et al., 2020). Risk factors are multifactorial, often featuring psychological stressors, trauma, and childhood adversity (Goleva et al., 2020; Hallett et al., 2022). Prevalence estimates range from 2 to 33 cases per 100,000 (Kanemoto et al., 2017) and incidence has been estimated at 4.9 per 100,000/year (Duncan et al., 2011). Women account for approximately 75% of cases (Goldstein et al., 2020).

At present, there exists a variety of different care pathways with no formalized consensus regarding management. This results in a lack of effective and consistent treatment options for people living with FS. Given the wide range of symptoms, high rates of mental health comorbidities, and misdiagnosis or lack of understanding of the diagnosis, people with FS are often overmedicated, which may exacerbate symptoms (Bennett et al., 2021). Psychological intervention (e.g., cognitive behavioral therapy (CBT), psychodynamic therapy, mindfulness approaches) is the current treatment of choice and may be associated with a reduction in seizures and improved function (Carlson and Nicholson Perry, 2017; Hingray et al., 2018). A recent multisite randomized controlled trial compared the addition of CBT to standardized medical care among 368 adults with FS and found no significant difference in seizure frequency at follow-up. However, the addition of CBT was associated with improvements in secondary outcomes such as psychosocial functioning and treatment satisfaction (Goldstein et al., 2020). This highlights the complexity associated with measuring outcome significance within treatment paradigms, further emphasizing the need for research and novel approaches to better understand what constitutes a successful intervention.

Ketamine, a glutamate receptor antagonist that selectively blocks the N-methyl-D-aspartate (NMDA) receptor, has been used as an anesthetic since 1964 and has a well-established safety and efficacy profile (Li and Vlisides, 2016). Following mounting evidence for ketamine as an antidepressant agent in the late 1990s and early 2000s, rapid progress has been made in the application of ketamine and ketamine-assisted therapy (KAT) for treating a broad range of psychiatric conditions (Berman et al., 2000; Krystal et al., 2019). This includes development of the S-enantiomer ketamine nasal spray, esketamine, approved by the United States FDA in 2019 for treatment-resistant depression. A recent systematic review of ketamine for treating mental health disorders highlights the robust, rapid, and typically short-lived antidepressant effects, with further evidence signaling applicability to conditions such as suicidality, anxiety, posttraumatic stress disorder (PTSD), obsessive–compulsive disorder, eating disorders, and substance use disorders (Walsh et al., 2022). While less robust than the research on depression, a growing body of evidence, including from randomized clinical trials, lends support to utilizing ketamine in the treatment of PTSD, particularly given that effective pharmacological treatments for PTSD are limited (Albott et al., 2018; Feder et al., 2021). The route of administration (i.e., oral/sublingual, intramuscular, intravenous, intranasal), constituent molecules [(R)-, (S)- or racemic ketamine] and whether therapeutic support is offered are key considerations. In general, KAT closely adheres to current models of psychedelic-assisted therapy that place emphasis on an integrated therapy model with inwardly directed, context-specific focus using eyeshades and music delivered in a comfortable setting, as well as preparatory and integrative psychotherapy support surrounding the treatment sessions.

Here we describe the case of a woman with FS and a history of multiple unsuccessful treatments. After 3 weeks of KAT and 20 weeks of ketamine maintenance with integrative psychotherapy, she experienced substantial improvements in her seizures and self-reported symptoms of depression, trauma, and functional wellbeing.

## Case presentation

We present a 51-year-old female with a medical history significant for major depressive disorder (MDD), PTSD and fibromyalgia. She does not have a history of epilepsy. She grew up in a home with caregivers who were dependent on alcohol and experienced significant neglect and abuse as a child. She tried dialectical behavioral therapy (DBT) skills groups and antidepressants which were unsuccessful. She is married and a mother to a six-year-old daughter who was adopted after fostering and a five-year-old son born in 2017 *via* surrogate. In the past 5 years both of her parents and in-laws have passed away.

**Figure 1:**
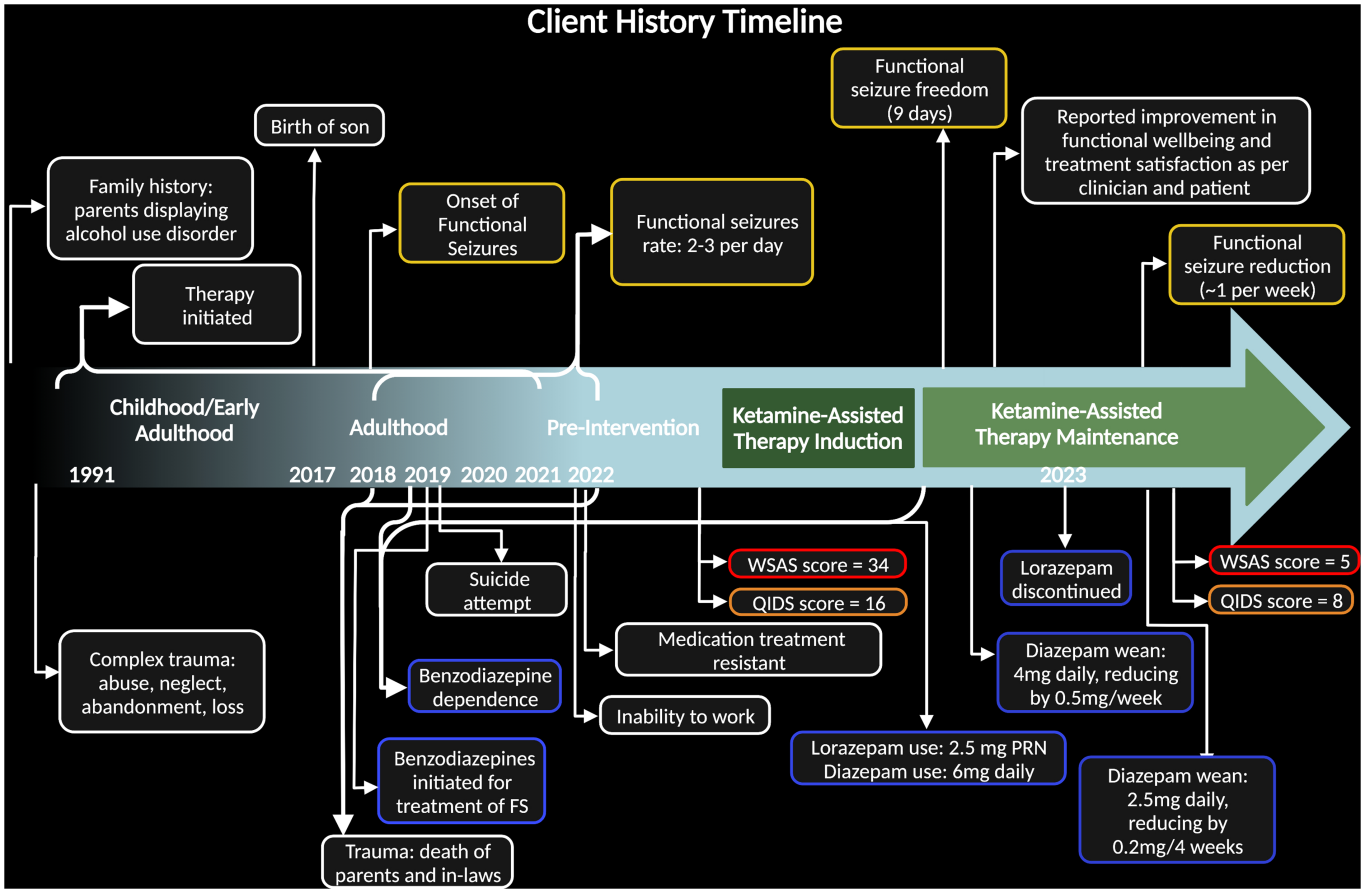
Timeline of case including client history highlighting relevant trauma, behavior, and symptoms. Dark green block designates time during ketamine-assisted therapy protocol and light green block designates ketamine maintenance. Dates along the bottom specify years of onset, with brackets expressing span of time. Yellow bubbles indicate function seizure severity, blue bubbles indicate benzodiazepine use, and red/orange bubbles emphasize pre- and post- case depression measures [Quick Inventory of Depressive Symptomatology (QIDS) measure and Work and Social Adjustment Score (WSAS)].

Beginning in 2018, the patient began experiencing seizure-like episodes occasionally associated with feelings of dissociation (Figure 1). By October 2022, these episodes increased to up to 3 times/day, each lasting approximately 30 min in duration. In the 7-month period prior to starting KAT, the patient’s seizures were debilitating to the point at which she was unable to continue her work as an aesthetician. The patient describes the episodes onsetting with an aura of nausea then developing into receptive aphasia with retention of audition and general awareness. Outward signs consisted of behavioral arrest, drooling, tearing and an inability to speak or move despite what appears like voluntary effort to observers. Some episodes manifested as tonic contraction of her toes, an inability to swallow, and repetitive extension-flexion movements of her neck.

The patient was evaluated for epilepsy and had several ambulatory EEGs which were all normal. An MRI was normal and was specifically absent of any structural changes in the fronto-temporal regions, mesial temporal area and temporal lobes bilaterally. In October 2021, an EEG captured one of her seizures and there was no electrographic correlate, suggesting a FS diagnosis. Our clinical team referred her to a community-based FS program, but she could not meet the demands of the program and never enrolled. As such, the patient did not receive FND- or FS-specific psychotherapy.

Prior to commencing KAT with our clinical team, the patient was seen by several other therapists and providers who attempted pharmacological approaches. Since 2019, the patient has been taking valproic acid (275 mg orally, twice a day) for mood stabilization, and diazepam (6 mg orally per day) for seizure control and sleep. She was also prescribed lorazepam as needed (0.5 mg sublingual) for seizure control; however, benzodiazepines are not a recognized treatment for FS. Her lorazepam use became extensive, increasing to 1.5 mg with every sensation of seizure onset, ranging, on average, from 1 to 3 times daily. Subsequently, she developed a benzodiazepine dependance and had a suicide attempt with an overdose of benzodiazepines in late 2019 (Figure 1).

In October 2022, the patient was self-referred with support/referral from her primary care physician to begin KAT as an alternative option at the Neurology Centre of Toronto (NCT) by Numinus Wellness. The clinical care team at NCT included a neurologist/epileptologist with FND and FS experience in adults and children; an intensive care unit nurse; a psychologist with clinical experience in trauma and somatization disorders; and a neurology physician assistant with clinical experience in epilepsy and FND. The Ontario Health Insurance Plan (OHIP) covered all publicly insured aspects of the treatment program (e.g., MD visits); however, the patient paid for uninsured components (e.g., psychotherapy and nursing care).

The KAT protocol at NCT for individuals with underlying neurological disorders has a standard, yet flexible framework. The framework includes group-led, virtual education sessions; medical, neurological, and psychological screening; preparatory psychotherapy sessions; weekly KAT sessions accompanied by the treating therapist [dose ranges from 50–300 mg sublingual (SL) and 15–60 mg intranasal (IN)]; post-treatment day integration sessions and evaluations; and closing medical and psychological assessments with ongoing integration therapy and individualized determination for maintenance ketamine therapy. Patient-specific treatment plans are determined in a weekly, multidisciplinary meeting of all team members who discuss and review the clinical findings and interpretations of the care team, much like Tumor Board Rounds or Epilepsy Surgery Rounds in hospital-based settings.

**Figure 2:**
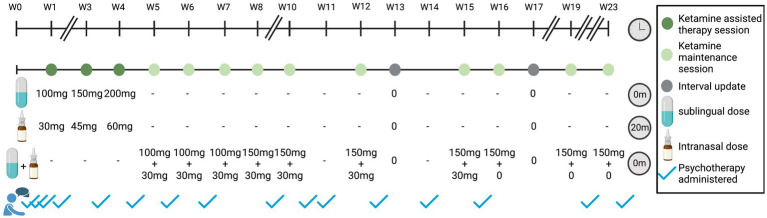
Ketamine-assisted therapy and maintenance over time. Timeline of treatment shown in weeks. Dark green indicates a ketamine-assisted therapy session, light green indicates ketamine maintenance session, gray indicates an interval update session, and blue checkmarks outside of dosing sessions indicate psychotherapy sessions. The standard NCT ketamine protocol consists of three 90-min preparatory sessions, three KAT sessions, and three 75-min integration sessions (1 day after KAT session). Maintenance sessions included treatment with ketamine overseen by a nurse with no therapist present. Sixty-minutes psychotherapy sessions followed as per the treating therapist recommendation. Breaks in timeline indicate weeks without any visits. For KAT treatment sessions, ketamine was administered sublingually (SL) at the beginning of the visit, and intranasally (IN) as a supplemental dose at 20 min. For maintenance sessions, the SL and IN dose are given at the beginning of the visit, with no supplemental dose. Interval update sessions include a phone check-in with the nurse.

In line with the standard NCT protocol for KAT, the patient underwent three 90-min preparatory sessions, three ketamine sessions with increasing doses (130–260 mg total of combined SL and IN routes; IN dose administered as a supplemental dose 20 min after SL administration, in accordance with patient comfort; Figure 2). Each ketamine session was followed by a 75-min integration session the following day. After the three initial KAT sessions, the patient received ongoing ketamine maintenance along with 60-min integration psychotherapy 1–10 days following dosing day. At the time of writing this report, the patient completed 10 ketamine maintenance sessions with increasing intervals between the sessions and variable total doses of 130–180 mg as combined SL and IN routes administered in parallel. Two more monthly maintenance sessions are planned and then ketamine will be discontinued while psychotherapy will be maintained.

**Figure 3:**
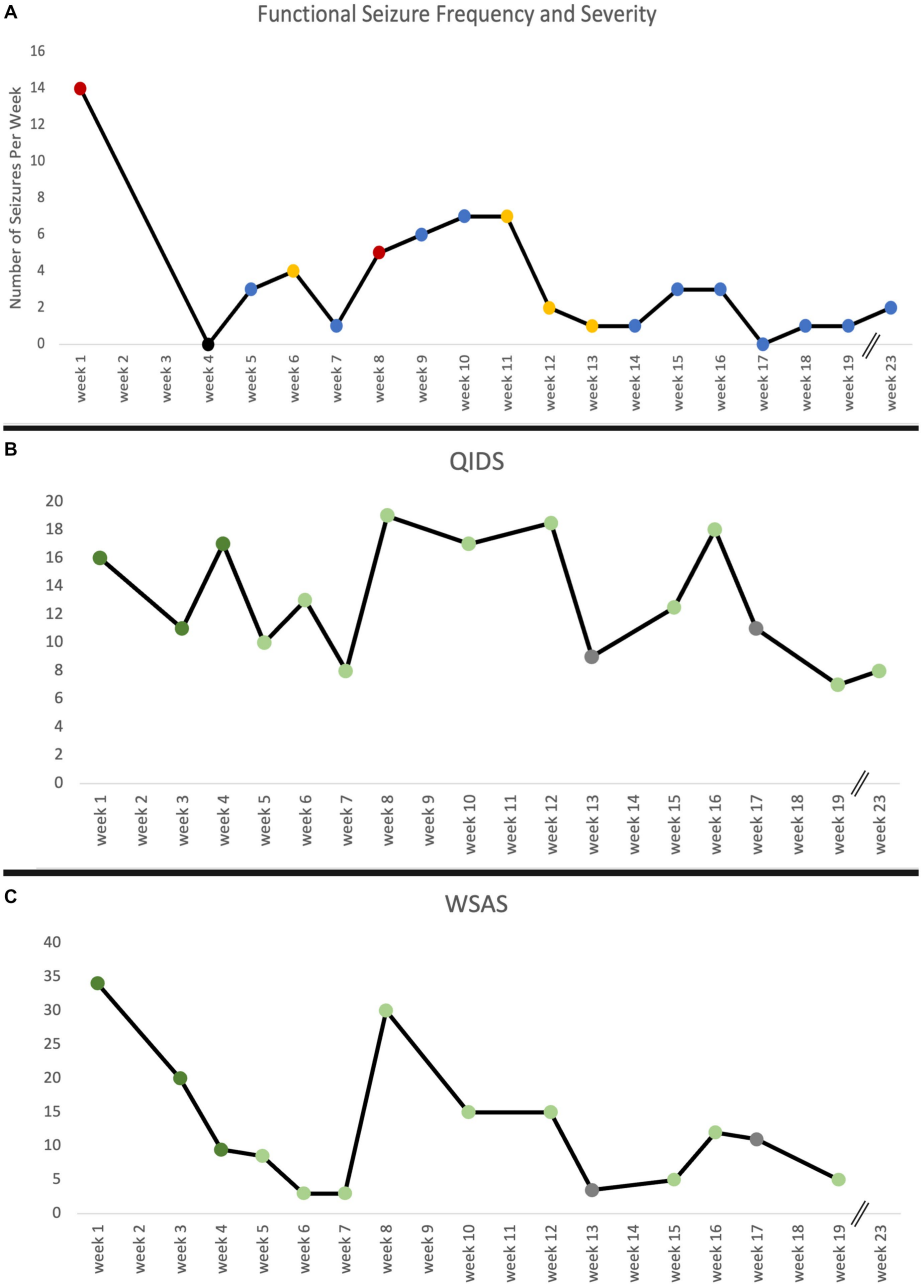
**(A)** Functional seizure frequency reported in number of seizures per week over the course of treatment and maintenance sessions as reported in participant seizure diary. Red indicates strong seizure severity, blue indicates mild seizure severity, and yellow indicates a mix of both types of seizure severity. No data on seizure frequency week 2 and 3 are due to missed data collection. **(B)** The Quick Inventory of Depressive Symptomatology (QIDS) and **(C)** Work and Social Adjustment Score (WSAS) measure over time. The QIDS and WSAS were administered at every treatment, maintenance, and interval update visit. Dark green indicates a ketamine-assisted therapy session, light green indicates ketamine maintenance session, and gray indicates an interval update session. No data on WSAS week 23 is due to missed measure administration. Breaks in timeline indicate weeks without any visits.

Following three KAT sessions, the patient experienced an immediate, significant reduction in the frequency of her seizures and went 9 consecutive days seizure-free. At the time of writing this report, the patient experienced two minor seizures in the last 3 weeks as compared to 7–21 per week prior to KAT (Figure 3). The seizures have also become less disabling, and she reports having the ability to “stop” some seizures from developing. Tapering of diazepam was an initial treatment goal and, at her request, began by the third treatment session. She is currently taking 1 mg each morning and 1.5 mg each night—a 58% dose reduction. She no longer uses lorazepam as needed (PRN) for seizure control which is a significant change from previous use of 1.5–4.5 mg daily prior to starting KAT.

Overall, FS reduced in both frequency and severity following KAT, and she experienced significant improvements in day-to-day function, quality of life and depression as captured by the Work and Social Adjustment Score (WSAS; 85% reduction) and Quick Inventory of Depressive Symptomatology (QIDS; 50% reduction). Prior to KAT, WSAS and QIDS scores were clinically significant and dropped to levels of insignificance following the latest maintenance session (Figure 3). The Patient Health Questionnaire (PHQ-9), Generalized Anxiety Disorder-7 (GAD-7), and PTSD Checklist DSM 5 (PCL-5) were administered after KAT and prior to maintenance sessions. Resultant scores have remained stable and below clinically relevant thresholds (Supplementary Table 2).

The patient had a typical FS at the end of her last treatment session. It lasted approximately 45 min and she returned to baseline with fatigue and no other concerns. She did not experience any other significant somatic nor psychological side effects associated with KAT and ketamine treatments. She described ketamine as “an existential experience” where she was “propelled into the galaxy” which made her feel like she gave herself permission to trust herself and her own feelings. Importantly, it allowed her to process and access past trauma without having a seizure. Exploring her seizures in therapy revealed that they were often triggered by reminders of past traumatic experiences or intense emotions. Therefore, processing and integrating trauma became an important target of therapy. She had not been able to achieve this successfully with other medical interventions nor in past therapy sessions as attempts to process trauma would trigger dissociative seizures. Notably, the patient experienced a FS during a preparatory psychotherapy session when asked to disclose her developmental history.

## Discussion

To our knowledge, this is the first case report describing the use of ketamine to treat FS. Following the KAT protocol as described, the patient experienced a substantial reduction of seizures, lower scores on co-morbid mental health assessments, an improved ability to disclose and process past trauma, and return to instrumental activities of daily living with a marked improvement in quality of life. Notably, the patient had multiple previous unsuccessful medical and therapy treatments.

### Large-scale brain networks and connectivity

Several models have been put forward to explain the pathophysiology of FS, yet much remains unknown (Baslet, 2011; Brown and Reuber, 2016; Kanemoto et al., 2017; Szaflarski and LaFrance, 2018; Goleva et al., 2020; Hallett et al., 2022). Functional neuroimaging data have shown that FS are associated with abnormalities in large-scale brain network connectivity patterns, and ketamine has been shown to rapidly change network dynamics (Szaflarski and LaFrance, 2018; Demchenko et al., 2022). Therefore, we hypothesized that KAT could be a potential intervention for our patient. Her resultant positive outcomes together with the recent case report published by Vendrell-Serres et al. utilizing ketamine without psychotherapy to successfully treat functional paralysis (Vendrell-Serres et al., 2021) suggests that further research into ketamine’s effects on neuronal network connectivity in the context of FS and other functional neurological presentations should be explored.

### Brain networks known to be involved in FS and ketamine

The discrete characterization of abnormalities within neuronal networks for FS is undefined. In an uncontrolled case series of seven patients with FS, targeted repetitive transcranial magnetic stimulation (rTMS) directed at the right temporoparietal junction (TPJ)—a brain region within the default mode network (DMN)—resulted in improved seizure control (Peterson et al., 2018). Connectivity alterations in this brain region have been associated with an abnormal sense of agency and have been implicated mechanistically to explain functional somatic symptoms including FS (Hallett et al., 2022). However, research comparing rTMS to non-cortical stimulation control in patients with FND yields insignificant differences, suggesting that although the TPJ may be involved, TMS may not be the sufficient neuromodulator that affects cortical functioning (Garcin et al., 2017). The disentangling of mechanistic FND relief through cognitive-behavioral effects versus neuromodulation remains uncertain.

Determining which brain regions within large-scale networks are altered by ketamine is an area of active research. Networks that demonstrate both abnormal connectivity patterns in FS and undergo dynamic change in response to ketamine include the sensorimotor cortex and the DMN (Baslet, 2011; van der Kruijs et al., 2012; Szaflarski and LaFrance, 2018), making the right TPJ an overlapping potential candidate brain region of interest that should be further explored as a structural biomarker and/or an area to target treatment.

At the molecular level, ketamine exerts its primary action *via* NMDA receptor antagonism at GABAergic interneurons (Krystal et al., 2019; Kohtala, 2021). The downstream effects are well described elsewhere (Stahl, 2021), and result in rapid synaptogenesis thought to explain the observed antidepressant effects of ketamine (Abdallah et al., 2017). It is possible that ketamine’s mechanism of action may provide the synaptic efficiency required to unlearn reinforced feedback loops in brain regions thought to potentiate FS (Hallett et al., 2022; Holmes et al., 2022). FS carry a high degree of comorbidity with MDD (Goleva et al., 2020) and MDD was an associated feature in our patient’s presentation. As such, an understanding of how these two systems interact may provide a model to help clarify ketamine’s positive effects.

### The potential benefit of KAT in the treatment of FS

Despite more than 20 years of ketamine data demonstrating a fast-acting antidepressant effect, lack of a sustained response is consistently reported (Kohtala, 2021). The psychedelic-assisted therapy model has demonstrated both short and long-term improvements in MDD and PTSD in clinical trials utilizing psilocybin and MDMA—which, like ketamine, also promote alteration of brain network connectivity (Carhart-Harris et al., 2021; Davis et al., 2021; Mitchell et al., 2021; Gukasyan et al., 2022; Singleton et al., 2023).

Ketamine-assisted therapy provides integrative therapy based on the psychedelic-assisted therapy model described in these clinical trials and others (Reiff et al., 2020). This approach may optimize patient outcomes by more effectively treating comorbid psychological conditions and providing an opportunity to produce long-term change beyond what has been reported in the literature regarding ketamine-only infusions for depression and other mental health conditions (Albott et al., 2018; Dore et al., 2019; Walsh et al., 2022).

Most presentations in FS are associated with comorbid psychological conditions that may also be present as preceding risk factors in developmental histories of patients (Baslet, 2011; Brown and Reuber, 2016; Goleva et al., 2020). The addition of psychotherapy in the KAT model with a focus on treating these conditions may be one of the key factors for a successful treatment paradigm for FS. Indeed, the patient’s FS may have improved secondary to KAT treating her depression and/or PTSD.

Our patient’s pre-treatment QIDS scores represented severe depression. These precipitously dropped in the first few weeks—representative of the rapid, early-onset effect—then returned to pre-treatment baseline levels from week 8 to 13 mirroring the lack of a sustained response that has been described in the literature for ketamine-only infusions. As the protocol continued, however, scores began to fall again up until the writing of this report with a sustained outcome representative of scores in the mild depression range and associated similar decreases in other markers of mental health (Figure 3; Supplementary Tables 1, 2). Additionally, seizure frequency appeared to fluctuate with QIDS and WSAS scores as shown in Figure 3.

It is possible that the initial antidepressant effect may have been rescued by the psychotherapeutic component of the KAT model as integrative psychotherapy remained consistent over several weeks despite ketamine dosing decreasing in frequency and amount. In this model, the subjective experiences explored through therapy may have sustained ketamine’s early, pharmacologically mediated improvements as psychotherapy promotes openness to exploring difficult emotions or experiences and can enhance the patient’s ability to meaningfully engage. Given literature describing the relevant impact of emotion in triggering FS, our patient’s ability to process her previous trauma and gain critical insight supports the current evidence (Baslet, 2011; Brown and Reuber, 2016; Goleva et al., 2020). Additionally, awareness of the etiology of FS, including past trauma, can lead to relief of symptoms alone (Hallett et al., 2022). Therefore, while ketamine’s primary action is as an NMDA receptor antagonist, it may help to reorganize impaired structural network connectivity while priming flexibility within these networks for concurrent psychotherapy to facilitate sustained change as the direct pharmacological effect of ketamine wears off and altered brain networks stabilize (van der Kruijs et al., 2012; Szaflarski and LaFrance, 2018).

### What significant research and clinical questions are generated by our report?

The striking results observed with our patient generate many important and specific research questions regarding a potential mechanism that, to date, has remained largely unexplored. Some of these questions include: is ketamine’s positive effect on FS mediated through brain network reorganization and, if so, which large-scale brain networks are involved? Do these networks represent clinical neural correlates for FS and/or the effective regions responsible for symptom manifestation? Should treatment be directed at the networks comprising the neural correlates and/or the effective regions? Does the addition of psychotherapy in the KAT model enhance outcomes and longer-term improvements of FS and, if so, how and why?

## Conclusion

Findings suggest that KAT may be helpful in treating FS in patients with comorbid depression and PTSD, yet further research is needed. Given ketamine’s established safety profile, KAT offers a potentially beneficial alternative therapy for FS, especially in refractory conditions with long-standing symptoms and impairment of daily function. In general, a more comprehensive understanding of ketamine’s effect on large-scale brain networks, how these networks are affected in FS and the role of psychotherapy is necessary to advance and strengthen the understanding of KAT’s applicability for FS and, perhaps, FND more broadly.

## Data availability statement

The original contributions presented in the study are included in the article/Supplementary material, further inquiries can be directed to the corresponding author.

## Ethics statement

Ethical review and approval was not required for the study on human participants in accordance with the local legislation and institutional requirements. The patients/participants provided their written informed consent to participate in this study. Written informed consent was obtained from the individual(s) for the publication of any potentially identifiable images or data included in this article. Written informed consent was obtained from the participant prior to the publication of this case report.

## Author contributions

EA, EO, and EL contributed to the conception of the case report. EA, EO, AJ, and EL wrote sections of the manuscript. EA, EO, AJ, AK, KM, CN, PT, and EL contributed to the manuscript and approved the final version. All authors contributed to the article and approved the submitted version.

## Funding

EA is supported by a Canadian Institutes of Health Research (CIHR) postdoctoral award. Funds for open access fees are provided by Numinus Wellness Inc.

## Conflict of interest

EA was a part-time consultant to Numinus Wellness Inc. and AJ, AK, KM, PT, CN, and EL were employed by the company Numinus Wellness Inc.

The remaining author declares that the research was conducted in the absence of any commercial or financial relationships that could be construed as a potential conflict of interest.

## Publisher’s note

All claims expressed in this article are solely those of the authors and do not necessarily represent those of their affiliated organizations, or those of the publisher, the editors and the reviewers. Any product that may be evaluated in this article, or claim that may be made by its manufacturer, is not guaranteed or endorsed by the publisher.
