# Supplementary material for: Case report: Improvement in refractory functional seizures, depression, and quality of life with ketamine-assisted therapy
Source: Front Neurosci. 2023 Jun 12;17:1197409. doi: 10.3389/fnins.2023.1197409 (PMC10291615; doi:10.3389/fnins.2023.1197409)
Supplement: Supplementary file 1 [file Data_Sheet_1.docx]

Supplementary Material

Case Report: Improvement in refractory functional seizures, depression, and quality of life with ketamine-assisted therapy

Elena Argento PhD^1,2*^, Egiroh Omene MD, FRCPC^3†^, Alexandria H Jaeger MSc^1†^, Angela Kertes PhD, C.Psych^3,4^, Kaitlyn A Mitchell MSc, RN^3^, Candace Necyk PhD^1^, Paul Thielking MD^5^, Evan Cole Lewis MD, FRCPC^3^

^1^Numinus Wellness Inc., Vancouver, BC, Canada

^2^British Columbia Centre on Substance Use, Faculty of Medicine, University of British Columbia, Vancouver, BC, Canada

^3^Neurology Centre of Toronto by Numinus, Toronto, ON, Canada

^4^North Toronto Psychology, Toronto, ON, Canada

^5^Numinus Wellness, Draper, UT, USA

^†^These authors contributed equally to this work

*** Correspondence:**
Elena Argento, PhD, MPH
[bccsu-ea@bccsu.ubc.ca](mailto:bccsu-ea@bccsu.ubc.ca)

| **Session** | **Week** | | **WSAS** | | **QIDS** | **Warwick Edinburgh** | **Sublingual Dose (mg)** | **Intranasal Dose (mg)** | **Total Dose (mg)** |
| --- | --- | --- | --- | --- | --- | --- | --- | --- | --- |
| **Treatment 1** | Week 1 | | 34 | | 16 | 49 | 100 | 30 | 130 |
| **Treatment 2** | Week 3 | | 20 | | 11 | 54 | 150 | 45 | 195 |
| **Treatment 3** | Week 4 | | 9.5 | | 17 | 46 | 200 | 60 | 260 |
| **Maintenance 1** | Week 5 | | 8.5 | | 10 | 64 | 100 | 30 | 130 |
| **Maintenance 2** | Week 6 | | 3 | | 13 | 66 | 100 | 30 | 130 |
| **Maintenance 3** | Week 7 | | 3 | | 8 | 58 | 100 | 30 | 130 |
| **Maintenance 4** | Week 8 | | 30 | | 19 | 32 | 150 | 30 | 180 |
| **Maintenance 5** | Week 10 | | 15 | | 17 | 37 | 150 | 30 | 180 |
| **Maintenance 6** | Week 12 | | 15 | | 18.5 | 29.5 | 150 | 30 | 180 |
| **Interval Update** | Week 13 | | 3.5 | | 9 | 45 | 0 | 0 | 0 |
| **Maintenance 7** | Week 15 | | 5 | | 12.5 | 42 | 150 | 30 | 180 |
| **Maintenance 8** | Week 16 | | 12 | | 18 | 38 | 150 | 0 | 150 |
| **Interval Update** | Week 17 | | 11 | | 11 | 49 | 0 | 0 | 0 |
| **Maintenance 9** | | Week 19 | 5 | 7 | | 50 | 150 | 0 | 150 |
| **Maintenance 10** | | Week 23 | *n/a | 8 | | 54 | 150 | 0 | 150 |

**Supplementary Table 1:** Psychosocial Tests and Dosing during Treatment and Maintenance Sessions. Psychosocial measures administered at treatment, maintenance sessions and interval updates. Quick Inventory of Depressive Symptomatology (QIDS); Work and Social Adjustment Score (WSAS), Warwick Edinburgh. Dosing breakdown in milligrams (mg) by route of administration of ketamine for all sessions. Treatment session dosing was administered as a stack of sublingual (SL) and intranasal (IN) routes, administered in series with the IN as a supplement dispensed at 20 minutes. Maintenance session dosing was administered as a combination of SL and IN routes in parallel. *n/a as measure was not administered.

| **Session** | **Week** | **PHQ-9** | **GAD-7** | **PCL5** |
| --- | --- | --- | --- | --- |
| **Maintenance 1** | Week 5 | 3 | 5 | 11 |
| **Maintenance 4** | Week 8 | 16 | 13 | 29 |
| **Interval Update** | Week 13 | 4.5 | 4.5 | 6 |
| **Maintenance 7** | Week 15 | *n/a | 8 | 14 |
| **Maintenance 8** | Week 16 | 9 | 7 | 13.5 |
| **Interval Update** | Week 17 | 8 | 5 | 26 |
| **Maintenance 9** | Week 19 | 5 | 11 | 14 |
| **Maintenance 10** | Week 23 | 2 | 3 | 12 |

**Supplementary Table 2:** Psychosocial measures administered at maintenance sessions and interval updates. Personal Health Questionnaire-9 (PHQ-9); Generalized Anxiety Disorder-7 (GAD-7); PTSD Checklist DSM 5 (PCL-5). *n/a as measure was not administered.
